# Notch Activation Is Dispensable for D, L-Sulforaphane-Mediated Inhibition of Human Prostate Cancer Cell Migration

**DOI:** 10.1371/journal.pone.0044957

**Published:** 2012-09-07

**Authors:** Eun-Ryeong Hahm, Kumar Chandra-Kuntal, Dhimant Desai, Shantu Amin, Shivendra V. Singh

**Affiliations:** 1 Department of Pharmacology and Chemical Biology, and University of Pittsburgh Cancer Institute, University of Pittsburgh School of Medicine, Pittsburgh, Pennsylvania, United States of America; 2 Department of Pharmacology, Penn State Milton S. Hershey Medical Center, Hershey, Pennsylvania, United States of America; University of California Irvine, United States of America

## Abstract

D, L-Sulforaphane (SFN), a synthetic racemic analog of broccoli constituent L-sulforaphane, is a highly promising cancer chemopreventive agent with *in vivo* efficacy against chemically-induced as well as oncogene-driven cancer in preclinical rodent models. Cancer chemopreventive effect of SFN is characterized by G_2_/M phase cell cycle arrest, apoptosis induction, and inhibition of cell migration and invasion. Moreover, SFN inhibits multiple oncogenic signaling pathways often hyperactive in human cancers, including nuclear factor-κB, Akt, signal transducer and activator of transcription 3, and androgen receptor. The present study was designed to determine the role of Notch signaling, which is constitutively active in many human cancers, in anticancer effects of SFN using prostate cancer cells as a model. Exposure of human prostate cancer cells (PC-3, LNCaP, and/or LNCaP-C4-2B) to SFN as well as its naturally-occurring thio-, sulfinyl-, and sulfonyl-analogs resulted in cleavage (activation) of Notch1, Notch2, and Notch4, which was accompanied by a decrease in levels of full-length Notch forms especially at the 16- and 24-hour time points. The SFN-mediated cleavage of Notch isoforms was associated with its transcriptional activation as evidenced by RBP-Jk-, HES-1A/B- and HEY-1 luciferase reporter assays. Migration of PC-3 and LNCaP cells was decreased significantly by RNA interference of Notch1 and Notch2, but not Notch4. Furthermore, SFN-mediated inhibition of PC-3 and LNCaP cell migration was only marginally affected by knockdown of Notch1 and Notch2. Strikingly, SFN administration to Transgenic Adenocarcinoma of Mouse Prostate transgenic mice failed to increase levels of cleaved Notch1, cleaved Notch2, and HES-1 proteins *in vivo* in prostatic intraepithelial neoplasia, well-differentiated carcinoma or poorly-differentiated prostate cancer lesions. These results indicate that Notch activation is largely dispensable for SFN-mediated inhibition of cell migration, which should be viewed as a therapeutic advantage as Notch activation is frequent in human prostate cancers.

## Introduction

D, L-Sulforaphane (SFN), a synthetic racemic analog of broccoli-derived L-isomer (L-SFN), is a highly promising cancer chemopreventive agent with remarkable activity in preclinical animal models [1], [2]. Talalay and co-workers were the first to observe prevention of 9,10-dimethyl-1,2-benzanthracene-induced mammary cancer in rats by this compound [3]. Cancer chemopreventive efficacy of SFN or L-SFN was subsequently extended to other chemical carcinogenesis models. For example, SFN administration was shown to suppress azoxymethane-induced colonic aberrant crypt foci in rats [4]. Likewise, SFN treatment resulted in prevention of benzo[a]pyrene-induced forestomach cancer and inhibition of malignant progression of lung adenomas induced by tobacco carcinogen 4-(methylnitrosamino)-1-(3-pyridyl)-1-butanone in mice [5], [6]. More recent studies have utilized transgenic mouse models to establish chemopreventive efficacy of SFN against oncogene-driven cancers. For instance, dietary administration of 300 and 600 ppm SFN for 3 weeks to ApcMin/+ mice resulted in suppression of polyps in the small intestine in a dose-dependent manner [7]. Previous studies from our laboratory have shown that oral gavage of 6 μmol SFN (three times per week) beginning at 6–7 weeks of age significantly inhibited incidence and burden of prostatic intraepithelial neoplasia (PIN) and/or well-differentiated prostate cancer (WD) as well as pulmonary metastasis multiplicity in Transgenic Adenocarcinoma of Mouse Prostate (TRAMP) mice without causing any side effects [8]. Consistent with these data [8], 8-week old TRAMP mice fed with 240 mg of broccoli sprouts/mouse/day exhibited a significant decrease in prostate tumor growth in another study [9]. Furthermore, growth of PC-3 human prostate cancer cells xenografted in male athymic mice was retarded significantly by oral treatment with SFN [10].

Because of promising results in preclinical rodent models [3]–[8], [10] elucidation of the mechanism underlying cancer chemopreventive response to SFN has been the topic of intense research over the past decade. Mechanisms contributing to cancer chemoprevention by SFN include: inhibition of CYP2E1 [11], cell cycle arrest [12], [13], apoptosis induction [12], [14], suppression of angiogenesis [15], inhibition of histone deacetylase [16], protein binding [17], induction of phase 2 enzymes [18], epigenetic repression of *hTERT* [19], and inhibition of self-renewal of breast cancer stem cells [20]. Mechanistic studies using cultured cancer cells have also revealed SFN-mediated suppression of various oncogenic pathways often hyperactive in human cancers, including nuclear factor-κB, androgen receptor, Bcl-2, Bcl-xL, and signal transducer and activator of transcription 3 [14], [21]–[23]. While activation of signal transducer and activator of transcription 3 confers modest protection against SFN-induced apoptosis, mitochondria-derived reactive oxygen species (ROS) provide initial signal for apoptosis commitment in cancer cells exposed to this agent [14], [24].

The Notch signaling has been implicated in prostate cancer development and metastasis [25]–[28]. For example, a study involving tumor samples from 154 men showed overexpression of Jagged-1, a Notch ligand, in metastatic prostate cancer compared with localized cancer and benign prostate disease [26]. Likewise, Bin Hafeez et al. [27] found increased expression of Notch1 in prostate cancers. Moreover, knockdown of *Notch1* inhibited invasion of human prostate cancer cells in association with inhibition of matrix metalloproteinase-9 (MMP-9) and urokinase plasminogen activator [27]. Down-regulation of Notch1 and its ligand Jagged-1 has been shown to inhibit proliferation of prostate cancer cells [28]. The present study used cultured human prostate cancer cells (PC-3, LNCaP, and LNCaP-C4-2B), and dorsolateral prostate tissues from control and SFN-treated TRAMP mice [8] to determine the role of Notch1, Notch2, and Notch4 in anticancer effects of SFN.

## Results

### SFN Treatment Increased Levels of Cleaved Notch1, Cleaved Notch2, and Cleaved Notch4 in Cultured Human Prostate Cancer Cells

**Figure 1:**
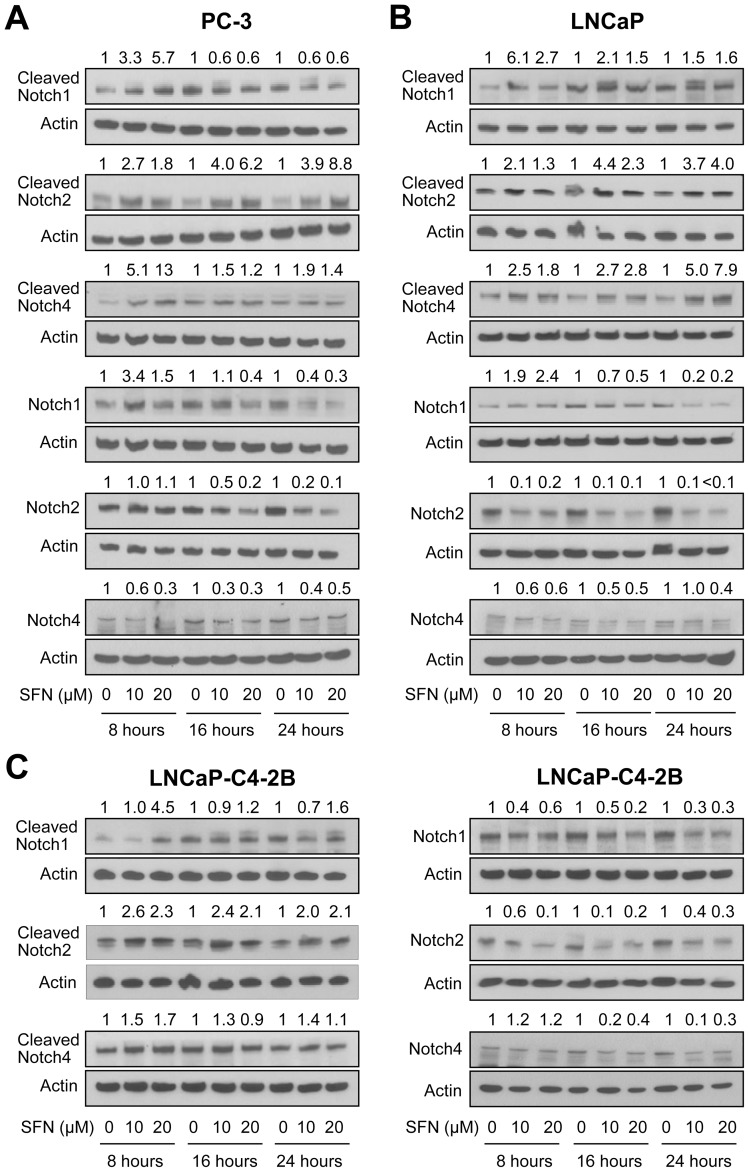
D, L-Sulforaphane (SFN) causes cleavage of Notch1, Notch2, and Notch4 proteins in PC-3 and LNCaP cells. Immunoblotting for cleaved and full-length Notch1, Notch2, and Notch4 using lysates from (A) PC-3, (B) LNCaP, and (C) LNCaP-C4-2B cells after 8-, 16-, or 24-hour treatment with DMSO or SFN (10 or 20 µM). Blots were stripped and re-probed with anti-actin antibody as a loading control. Immunoblotting for each protein was done at least twice using independently prepared lysates. Numbers above band represent changes in protein levels relative to corresponding DMSO-treated control.

Notch activation involves binding of the receptor to adjoining ligands followed by a conformational change within the receptor and Notch cleavage mediated by the γ-secretase complex at a site located within the Notch transmembrane domain [29]. The net outcome of these reactions is release of the Notch intracellular domain into the cytoplasm, which then translocates to the nucleus to regulate target gene expression [25], [29]. We used PC-3 (an androgen-independent human prostate cancer cell line lacking functional p53), LNCaP (an androgen-responsive human prostate cancer cell line with wild-type p53), and LNCaP-C4-2B (an androgen-independent variant of the LNCaP cell line) to study the role of Notch signaling in anticancer effects of SFN. Levels of cleaved Notch1, cleaved Notch2, and cleaved Notch4 proteins were increased markedly upon treatment with SFN in PC-3 (Fig. 1A), LNCaP (Fig. 1B), and LNCaP-C4-2B cells (Fig. 1C), albeit with different kinetics. For example, unlike LNCaP cells (Fig. 1B), cleavage of the Notch1 upon treatment with SFN was transient (increased cleavage seen only at 8 hour- time point) in PC-3 cells (Fig. 1A). Molecular basis for cell line-specific differences in Notch activation by SFN is not clear, but Notch activation by SFN was accompanied by a decrease in the levels of full-length Notch1, Notch2, and Notch4 in each cell line especially at the 16- and 24-hour time points (Fig. 1A–C). Notably, Notch2 was generally more sensitive to cleavage by SFN compared with Notch1 or Notch4 (Fig. 1A–C). Collectively, these results indicated that SFN treatment resulted in cleavage of Notch1, Notch2, and Notch4 in both androgen-independent and androgen-responsive human prostate cancer cells.

### Effects of Naturally-Occurring Analogs of SFN on Notch Activation in PC-3 and LNCaP Cells

**Figure 2:**
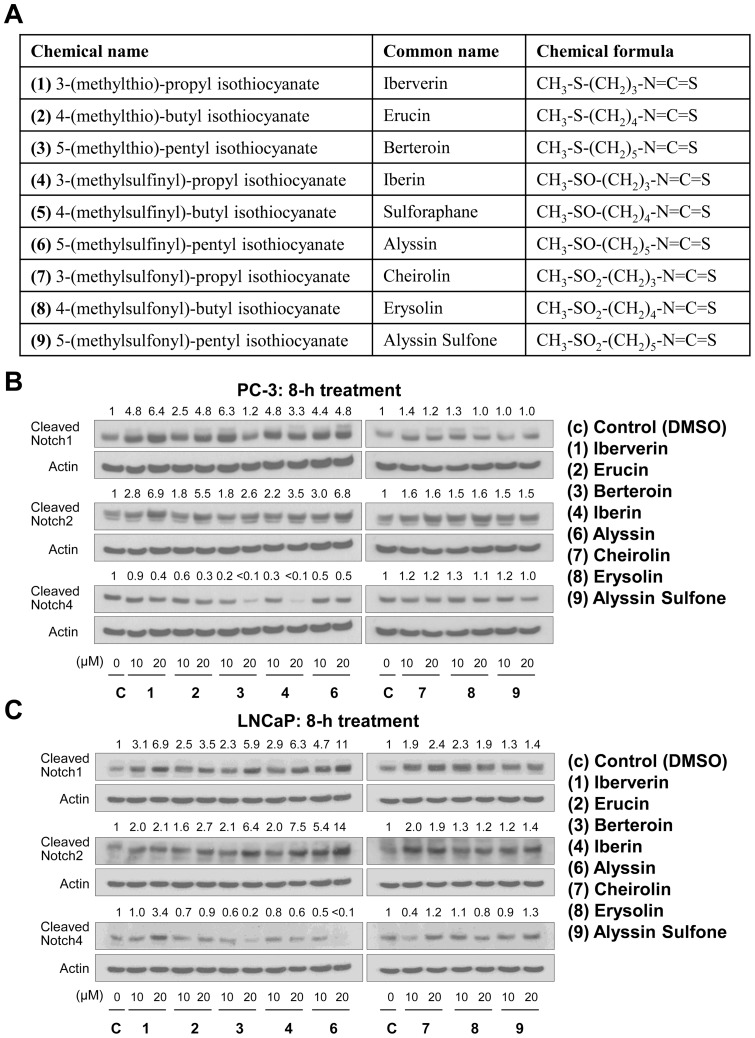
Effects of analogs of D, L-sulforaphane on cleavage of Notch isoforms. (A) Chemical names, common names, and chemical formulae of analogs used in the present study. Immunoblotting for cleaved Notch1, Notch2, and Notch4 using lysates from (B) PC-3, and (C) LNCaP cells after 8-hour treatment with DMSO or different analogs (10 or 20 µM). Blots were stripped and re-probed with anti-actin antibody as a loading control. Immunoblotting for each protein was done at least twice using independently prepared lysates. Numbers above band represent changes in protein levels relative to DMSO-treated control.

We used naturally-occurring thio-, sulfinyl-, and sulfonyl-analogs of SFN with different alkyl chain length (Fig. 2A) to determine if activation of Notch was unique to SFN. Eight-hour exposure of PC-3 (Fig. 2B) and LNCaP cells (Fig. 2C) to thio- (Iberverin, Erucin, and Berteroin) and sulfinyl-analogs (Iberin and Alyssin) resulted in cleavage of Notch1 and Notch2 (Fig. 2B, C). Furthermore, the thio- and sulfinyl-analogs were relatively more potent in causing cleavage of Notch1 and Notch2 in comparison with sulfonyl-analogs (Cheirolin, Erysolin, and Alyssin Sulfone) in both PC-3 (Fig. 2B) and LNCaP cells (Fig. 2C). Surprisingly, levels of cleaved Notch4 were decreased to varying extent upon treatment with thio- and sulfinyl-analogs but not sulfonyl-analogs in both cell lines. Collectively, these results indicated that the naturally-occurring thio- and sulfinyl-analogs of SFN were effective in causing cleavage of Notch1 and Notch2. On the other hand, Notch4 activation seems unique to SFN.

### Effect of SFN Treatment on Transcriptional Activity of Notch

**Figure 3:**
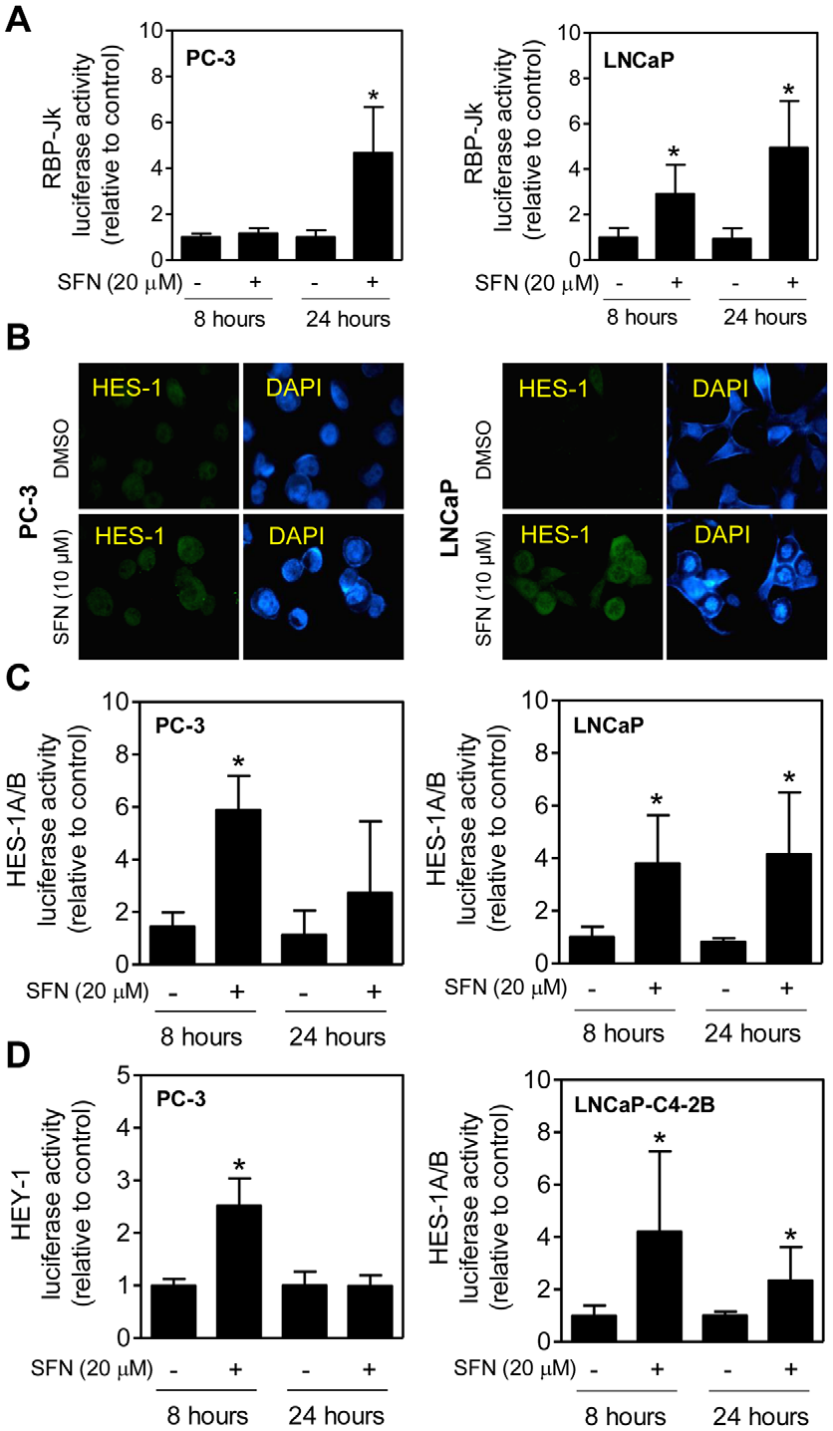
D, L-Sulforaphane (SFN) increases transcriptional activity of Notch in prostate cancer cells. (A) Effect of SFN treatment on RBP-Jk luciferase reporter activity (a measure of transcriptional activity of Notch) in PC-3 and LNCaP cells after 8- or 24-hour treatment with DMSO or 20 µM SFN. (B) Immunofluorescence microscopic images depicting nuclear levels of HES-1 protein in PC-3 and LNCaP cells after 24-hour treatment with DMSO or 10 µM SFN (×100 objective magnification). (C) HES-1A/B luciferase reporter activity in PC-3 and LNCaP cells after 8- or 24-hour treatment with DMSO or 20 µM SFN. (D) HEY-1 (PC-3 cells) or HES-1A/B (LNCaP-C4-2B cells) luciferase reporter activity after 8- or 24-hour treatment with DMSO or 20 µM SFN. In panels A, C, and D, results shown are mean ± SD (n = 6; combined data from two independent experiments each performed in triplicate). *Significantly different (*P*<0.05) compared with corresponding DMSO-treated control by Student's *t*-test (panels A, C, and D). Each experiment was performed twice.

We proceeded to test whether SFN-mediated cleavage of Notch1, Notch2, and Notch4 was accompanied by transcriptional activation of Notch using luciferase reporter assays. The RBP-Jk [C protein binding factor 1/Suppressor of Hairless/Lag1 (CBF1/Su (H)/Lag 1)] is a direct downstream modulator of Notch signaling [25], [29]. Exposure of PC-3 and LNCaP cells to 20 µM SFN for 8 and/or 24 hours resulted in a statistically significant increase in RBP-Jk luciferase reporter activity (Fig. 3A). Consistent with these results, the levels of nuclear HES-1, a downstream target of Notch, were increased upon 24-hour treatment of PC-3 and LNCaP cells with SFN compared with dimethyl sulfoxide (DMSO)-treated control cells (Fig. 3B). The luciferase activity associated with Notch target genes *HES-1A/B* and *HEY-1* (Fig. 3C, D) were also increased significantly upon treatment with 20 µM SFN in prostate cancer cells. Together, these observations indicated that SFN treatment caused transcriptional activation of Notch in cultured prostate cancer cells.

### Effect of RNA Interference of Notch1 on SFN-Mediated Inhibition of PC-3 and LNCaP Cell Migration

**Figure 4:**
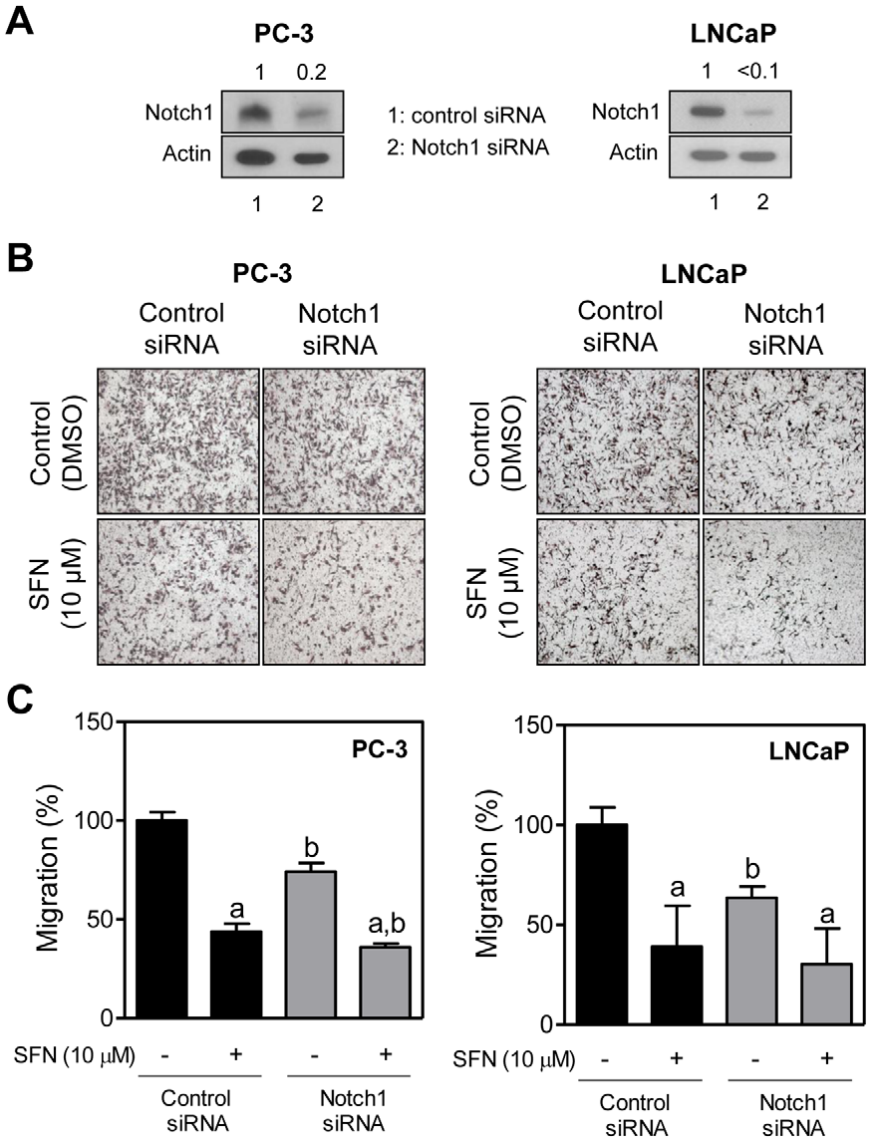
Effect of Notch1 silencing on D, L-sulforaphane (SFN)-mediated inhibition of PC-3 and LNCaP cell migration. (A) Immunoblotting for full-length Notch1 protein using lysates from PC-3 and LNCaP cells transiently transfected with a control (nonspecific) siRNA or a Notch1-targeted siRNA. (B) Representative images (Boyden chamber assay) depicting migration of PC-3 and LNCaP cells transfected with a control (nonspecific) siRNA or a Notch1-targeted siRNA and treated for 24 hours with DMSO or 10 μM SFN (×100 magnification). (C) Quantitation of PC-3 and LNCaP cell migration from data shown in panel B. Two to three fields on each filter were scored for cell migration under an inverted microscope. Data represent percent cell migration normalized to control siRNA-transfected cells treated with DMSO (mean ± SD, n = 6; combined data from two independent experiments each performed in triplicate). Significantly different (*P*<0.05) ^a^compared with respective DMSO-treated controls (cells transfected with control siRNA or Notch1-targeted siRNA), and ^b^between control siRNA transfected cells and Notch1-targeted siRNA transfected cells by one-way ANOVA followed by Bonferroni's multiple comparison test. Each experiment was performed twice.

Down-regulation of Notch1 was shown to inhibit prostate cancer cell migration and invasion [28]. We therefore designed experiments using PC-3 and LNCaP cells to determine the consequences of Notch1 activation on SFN-mediated inhibition of cell migration. The PC-3 and LNCaP cells transiently transfected with a Notch1-targeted siRNA exhibited 80% or greater decrease in the levels of full-length Notch1 when compared with the cells transfected with a control siRNA (Fig. 4A). RNA interference of Notch1 alone resulted in a significant decrease in PC-3 and LNCaP cell migration (Fig. 4B, C). However, the SFN-mediated inhibition of PC-3 and LNCaP cell migration was only marginally affected by knockdown of Notch1 (Fig. 4C). Based on these results, we conclude that Notch1 activation is largely dispensable for SFN-mediated inhibition of prostate cancer cell migration.

### Effect of Notch2 Protein Knockdown on SFN-Mediated Inhibition of PC-3 and LNCaP Cell Migration

**Figure 5:**
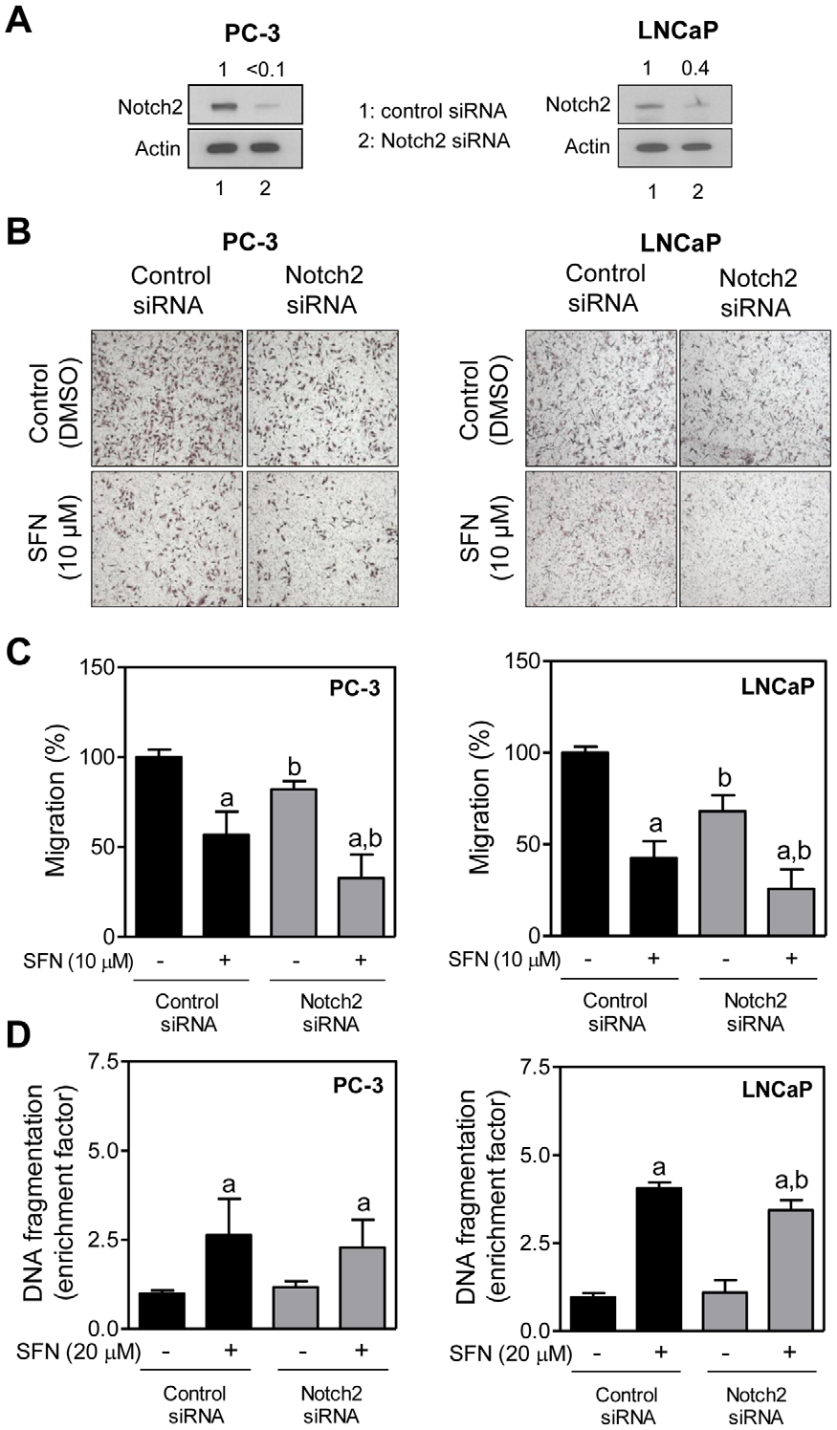
Effect of Notch2 knockdown on cellular responses to D, L-sulforaphane (SFN). (A) Immunoblotting for full-length Notch2 protein using lysates from PC-3 and LNCaP cells transiently transfected with a control (nonspecific) siRNA or a Notch2-targeted siRNA. (B) Representative images (Boyden chamber assay) depicting migration of PC-3 and LNCaP cells transfected with a control (nonspecific) siRNA or a Notch2-targeted siRNA and treated for 24 hours with DMSO or 10 μM SFN (×100 magnification). (C) Quantitation of PC-3 and LNCaP cell migration from data shown in panel B. Two to three fields on each filter were scored for cell migration under an inverted microscope. Data represent percent cell migration normalized to control siRNA transfected cells treated with DMSO (mean ± SD, n = 6; combined data from two independent experiments each performed in triplicate). (D) Analysis of histone-associated DNA fragment release into the cytosol in PC-3 and LNCaP cells transfected with a control (nonspecific) siRNA or a Notch2-targeted siRNA and treated for 24 hours with DMSO or SFN. Data represent enrichment of histone-associated DNA fragment release into the cytosol relative to control siRNA transfected cells treated with DMSO (mean ± SD, n = 6; combined data from two independent experiments each performed in triplicate). In panels C and D, significantly different (*P*<0.05) ^a^compared with respective DMSO-treated controls (cells transfected with control siRNA or Notch2-targeted siRNA), and ^b^between control siRNA transfected cells and Notch2-targeted siRNA transfected cells by one-way ANOVA followed by Bonferroni's multiple comparison test. Each experiment was performed twice.

We have shown previously that silencing of full-length Notch2 protein significantly decreases migration ability of both PC-3 and LNCaP cells [30]. We proceeded to test whether Notch2 activation by SFN affected its ability to inhibit PC-3 and LNCaP cell migration. Level of the full-length Notch2 protein was decreased by >90% and 60%, respectively, upon transient transfection of PC-3 and LNCaP cells with a Notch2-targeted siRNA when compared with the corresponding cells transfected with the control siRNA (Fig. 5A). Consistent with our earlier observations [30], knockdown of Notch2 protein alone reduced PC-3 and LNCaP cell migration (Fig. 5B, C). The SFN-mediated inhibition of PC-3 and LNCaP cell migration was modestly augmented by RNA interference of Notch2 (Fig. 5C). For example, PC-3 cell migration was inhibited by 43% and 18% (equals 59% inhibition), respectively, after 24-hour treatment of control siRNA transfected cells with 10 µM SFN and RNA interference of Notch2 alone. Migration of PC-3 cell was inhibited by 67% upon treatment with SFN in Notch2 silenced cells. These results suggested that Notch2 activation by SFN imparted marginal resistance against its inhibitory effect on cell migration.

O'Neill et al [31] have shown previously that Notch2 regulates apoptosis at least in MDA-MB-231 human breast cancer cells. Therefore, it was only logical to determine if Notch2 activation affected SFN-induced apoptosis. As shown in Fig. 5D, knockdown of Notch2 protein alone did not have any meaningful impact on histone-associated DNA fragment release into the cytosol, which is a well-accepted method for quantitation of apoptosis. The SFN-mediated histone-associated DNA fragment release into the cytosol was slightly abrogated upon RNA interference of Notch2 in LNCaP cells, but this differential did not reach statistical significance in the PC-3 cell line (Fig. 5D). Because of marginal effects and cell line-specific differences, we conclude that Notch2 activation has minimal impact on ability of SFN to induce apoptosis at least in prostate cancer cells.

### Notch4 Activation Was Dispensable for SFN-Mediated Inhibition of PC-3 Cell Migration

**Figure 6:**
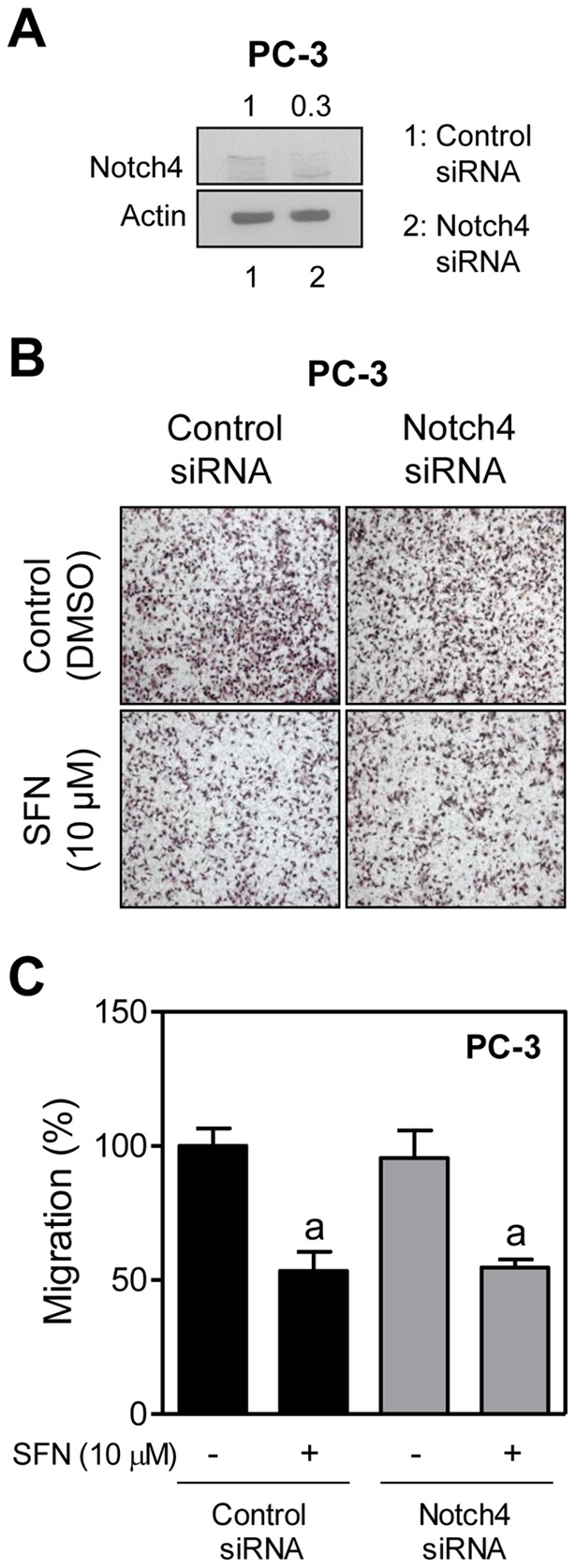
Notch4 is dispensable for D, L-sulforaphane (**SFN**)**-mediated inhibition of PC-3 cell migration.** (A) Immunoblotting for full-length Notch4 protein using lysates from PC-3 cells transiently transfected with a control (nonspecific) siRNA or a Notch4-targeted siRNA. (B) Representative images (Boyden chamber assay) depicting migration of PC-3 cells transfected with a control (nonspecific) siRNA or a Notch4-targeted siRNA and treated for 24 hours with DMSO or 10 μM SFN (×100 objective magnification). (C) Quantitation of PC-3 cell migration from data shown in panel B. Two to three fields on each filter were scored for cell migration under an inverted microscope. Data represent percent cell migration normalized to control siRNA-transfected cells treated with DMSO (mean ± SD, n = 6; combined data from two independent experiments each performed in triplicate). ^a^Significantly different (*P*<0.05) compared with respective DMSO-treated controls (cells transfected with control siRNA or Notch4-targeted siRNA) by one-way ANOVA followed by Bonferroni's multiple comparison test. Each experiment was performed twice.

Next, we proceeded to determine the role of Notch4 activation in SFN-mediated inhibition of cell migration using PC-3 cells. Level of full-length Notch4 protein was decreased by 70% upon transient transfection of PC-3 cells with a Notch4-targeted siRNA compared with cells transfected with the control siRNA (Fig. 6A). Unlike Notch1 (Fig. 4C) or Notch2 (Fig. 5C), RNA interference of Notch4 alone had minimal effect on PC-3 cell migration (Fig. 6B, C). Moreover, inhibition of PC-3 cell migration resulting from SFN exposure was not influenced by RNA interference of Notch4 (Fig. 6C). Thus activation of Notch4 was also dispensable for SFN-mediated inhibition of PC-3 cell migration at least in PC-3 cells. Similar studies using Notch4 siRNA were not performed in LNCaP cells.

### Imzmunohistochemical Analysis for Cleaved Notch1, Cleaved Notch2, and HES-1 Proteins in Dorsolateral Prostate Tissue Sections from Control and SFN-Treated TRAMP Mice

**Figure 7:**
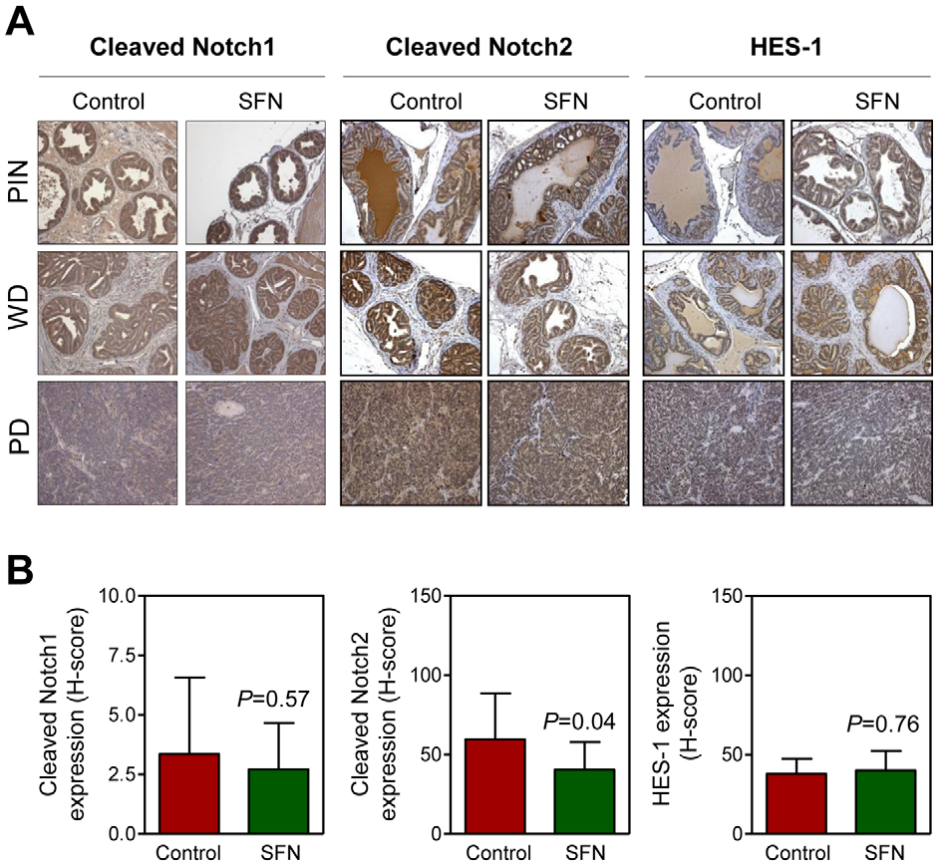
Effect of D, L-sulforaphane (SFN) administration on cleaved Notch1, cleaved Notch2, and HES-1 proteins *in vivo*. (A) Representative immunohistochemical images depicting expression of cleaved Notch1, cleaved Notch2, and HES-1 proteins in the prostatic intraepithelial neoplasia (PIN), well-differentiated prostate cancer (WD), and poorly-differentiated prostate cancer (PD) in the dorsolateral prostates from TRAMP mice of the indicated groups (×200 objective magnification). (B) Quantitation of expression (combined analysis in PIN, WD, and PD) is shown as H-score (mean ± SD; n = 5). Statistical significance was determined by Student's *t*-test.

We used archived paraffin-embedded prostate tissues from our previously completed TRAMP study [8] to determine *in vivo* effect of SFN administration on levels of cleaved Notch1, cleaved Notch2, and HES-1 proteins. Representative images for cleaved Notch1, cleaved Notch2, and HES-1 protein expression in the PIN, WD, and poorly-differentiated prostate cancer (PD) of control and SFN-treated TRAMP mice are shown in Fig. 7A. Surprisingly, SFN administration failed to increase levels of these proteins in PIN, WD, and PD. However, a modest but significant decrease in overall level of cleaved Notch2 (combined expression in PIN, WD, and PD) was discernible in the dorsolateral prostate of SFN-treated TRAMP mice compared with that of control TRAMP mice.

## Discussion

Notch signaling is quite complex involving interplay between four receptor (Notch1, Notch2, Notch3, and Notch4) and five ligands [Jagged1, Jagged2, Delta-like and ligands (Dll1, Dll3, and Dll4)] [25], [29]. Notch signaling is implicated in cell fate determination in embryonic and adult tissues [32], [33], and normal prostatic development as well as in pathogenesis of prostate cancers [25]. Overexpression of Jagged-1 was shown in metastatic prostate cancer compared with localized cancer and benign prostate disease [26]. Moreover, Notch1 knockdown was shown to inhibit the MMP-9 and invasion of PC-3 cells [27]. Down-regulation of Jagged1 has been shown to inhibit proliferation of prostate cancer cells [28]. RNA interference of Notch1 inhibits prostate cancer cell migration and invasion [28]. The present study shows that SFN treatment activates Notch signaling in human prostate cancer cell lines irrespective of the androgen-responsiveness. The SFN-mediated activation of Notch1, Notch2, and Notch4 is characterized by their cleavage and increased transcriptional activity. Activation of Notch1 and Notch2, but not Notch4, is not unique to SFN as some of its naturally-occurring analogs are effective in causing cleavage of both Notch1 and Notch2 in PC-3 and LNCaP cells. Consistent with literature data [28,] we also found that knockdown of Notch1 and Notch2 reduces migration of prostate cancer cells irrespective of the androgen-responsiveness. Surprisingly, Notch activation by SFN has minimal impact on its ability to inhibit prostate cancer cell migration. Specifically, knockdown of Notch1, Notch2 or Notch4 has no impact at all or only marginal effect on SFN-mediated inhibition of prostate cancer cell migration. These observations indicate that Notch activation is dispensable for SFN-mediated inhibition of prostate cancer cell migration.

Evidence continues to accumulate to indicate that structural differences in naturally-occurring isothiocyanates (ITC) can profoundly affect their activity. For example, autophagy induction by SFN serves to protect against apoptosis [34]. To the contrary, autophagy contributes to cell death induction by phenethyl isothiocyanate (PEITC) [35], which is a naturally-occurring constituent of watercress with structural similarity to SFN (i.e., presence of the ITC functional group). The present study provides yet another example to illustrate mechanistic differences in structurally-related ITC compounds (e.g., SFN and PEITC). We have shown previously that, unlike SFN (present study) Notch activation by PEITC impedes its inhibitory effect on prostate cancer cell migration [30]. These observations underscore caution in extrapolation of mechanistic results between structurally-different ITC compounds.

Activation of Notch2 by overexpression of its intracellular domain has been shown to promote apoptosis in MDA-MB-231 human breast cancer cells [31]. Because apoptosis induction is considered an important mechanism in cancer chemoprevention by SFN [12], [14], it was of interest to determine the consequences of Notch2 activation on proapoptotic response to SFN. Unlike MDA-MB-231 cells, knockdown of Notch2 has no impact on apoptosis in either PC-3 or LNCaP cells. Moreover, SFN-induced apoptosis was minimally affected by knockdown of Notch2 in PC-3 and LNCaP cells. Thus proapoptotic role of Notch2 may be a phenomenon unique to the MDA-MB-231 cells.

We have shown previously that SFN administration inhibits incidence and burden (affected area) of PIN and WD, but not PD, as well as pulmonary metastasis multiplicity in TRAMP mice [8]. Interestingly, SFN administration is unable to increase levels of cleaved Notch1, cleaved Notch2 or HES-1 *in vivo* in the dorsolateral prostate of TRAMP mice (present study). On one hand, these results suggest that prevention of prostate cancer by SFN in TRAMP mice is not related to Notch signaling. At the same time, the possibility that a more intense dosing regimen of SFN (e.g., higher concentrations and daily administration) may be required to elicit Notch activation *in vivo* can't be discarded. Observed discrepancy between *in vitro* and *in vivo* systems concerning effect of SFN on notch activation may also be related to tumor microenvironment. However, further work is needed to explore these possibilities.

In conclusion, the results of the present not only reveal *in vitro* and *in vivo* differences in effect of SFN on Notch activation but also indicate that activation of Notch1, Notch2, and Notch4 is largely dispensable for cellular responses to SFN (e.g., inhibition of cell migration or apoptosis) at least in human prostate cancer cells.

## Methods

### Ethics Statement

We used archived tissue sections from our previously published *in vivo* study [8] to determine the effect of SFN administration on expression of cleaved Notch1, cleaved Notch2, and HES-1 proteins. Use of mice and their care was approved by and in accordance with the University of Pittsburgh Institutional Animal Care and Use Committee guidelines.

### Reagents and Cell Lines

The SFN was synthesized as described by Conaway et al. [6], whereas its naturally-occurring analogs were purchased from LKT Laboratories (St. Paul, MN). Stock solutions of SFN and its analogs were prepared in DMSO, stored at −20°C, and diluted with fresh media immediately before use. The same volume of DMSO (final concentration <0.1%) was added to the control samples. Cell culture reagents such as fetal bovine serum, antibiotics mixtures, phosphate-buffered saline (PBS), and trypsin were purchased from Invitrogen-Life Technologies (Carlsbad, CA). Antibodies against cleaved Notch1, and full-length Notch2 were from Cell Signaling Technology (Danvers, MA); an antibody specific for detection of cleaved Notch2 was from EMD-Millipore (Billerica, MA); antibodies against full-length Notch1, Notch4 (this antibody detects both full-length and cleaved forms), and HES-1 were from Santa Cruz Biotechnology (Santa Cruz, CA); and anti-actin antibody was from Sigma-Aldrich (St. Louis, MO). Small interfering RNAs targeted against Notch1, Notch2, and Notch4 were purchased from Santa Cruz Biotechnology. A nonspecific control siRNA was purchased from Qiagen (Germantown, MD). PC-3 and LNCaP human prostate cancer cell lines were obtained from American Type Culture Collection (Manassas, VA) and cultured as described previously [14], [24]. LNCaP-C4-2B cell line was purchased from UroCor and maintained as suggested by the supplier.

### Immunoblotting

Whole cell lysates were prepared as described [36] and subjected to sodium dodecyl-sulfate polyacrylamide gel electrophoresis [36], [37]. The wet-transferred membrane was incubated with appropriate primary and secondary antibodies and the immunoreactive bands were detected using enhanced chemiluminescence method. Multiplexing or stripping/re-probing was performed for some proteins in some experiments.

### Luciferase Reporter Assay

The transcriptional activity of Notch was determined using a Cignal RBP-Jk luciferase reporter kit (SABiosciences-Qiagen) following the supplier's recommendations. For determination of the HES-1A/B and HEY-1 luciferase activity, we used pGL2-HES-1A/B and pGL2-HEY-1 plasmids generously given to us by Dr. Kimberly E. Foreman (Department of Pathology, Loyola University Medical Center, Maywood, IL). The transiently co-transfected cells with firefly luciferase plasmid and renilla luciferase using FuGENE6 (Roche Applied Science, Indianapolis, IN) were treated with DMSO (control) or SFN for 8 or 24 hours. Luciferase activity was measured by Dual-Luciferase Reporter Assay System (Promega, Madison, WI) following the manufacturer's protocols. Relative luciferase activity, the ratio of firefly-to-renilla luciferase activity, was normalized against protein concentration.

### Immunocytochemistry for HES-1

Cells grown on coverslips for overnight were treated with DMSO (control) or SFN for 24 hours, fixed with 2% paraformaldehyde for 1 hour at room temperature, and permeabilized using 0.5% Triton X-100 for 5 minutes. Cells were then blocked with PBS supplemented with 0.5% bovine serum albumin and 0.15% glycine for 1 hour and then incubated with anti-HES-1 antibody at 4°C for overnight. Next day, cells were treated with 2 μg/mL of Alexa Fluor-488-conjugated secondary antibody for 1 hour at room temperature. After washing with PBS cells were treated with 50 ng/mL of 4′,6-diamidino-2-phenylindole for 7 minutes at room temperature to stain nuclear DNA. Cells were visualized under a fluorescence microscope.

### RNA Interference

Cells were transfected at ∼50% confluency with 200 nmol/L of Notch1, Notch2, and Notch4-targeted siRNA or a nonspecific control siRNA using Oligofectamine (Invitrogen-Life Technologies). Twenty-four hours after transfection, the cells were treated with DMSO (control) or SFN for 24 hours. Subsequently, the cells were collected and processed for western blotting as well as for assessments of cell migration and apoptosis.

### Measurement of Apoptosis and Cell Migration

Histone-associated DNA fragment release into the cytosol was quantified using an ELISA kit (Roche Applied Science) according to the supplier's instructions. Transwell Boyden chambers containing 8.0 μm pore size polycarbonate filter (Corning-Life Sciences, Lowell, MA) were used to determine cell migration. The cells transfected with a nonspecific control siRNA or Notch1-, Notch2-, or Notch4-targeted siRNAs were used for cell migration assay described by us previously [30], [38].

### Immunohistochemistry

Tissue sections were immunostained with antibodies against cleaved Notch1, cleaved Notch2, and HES-1 as described by us previously for other proteins [37]. Immunohistochemical analysis was performed using Nuclearv9.1 algorithm of Aperio ImageScope software (Aperio Technologies, Vista, CA). Results are presented as H-score.

### Statistical Analysis

All statistical tests were performed using GraphPad Prism v.4.03 software (GraphPad Software, La Jolla, CA).
